# Negative Impact of Olanzapine on ICU Delirium Resolution: An Emulated Clinical Trial

**DOI:** 10.3390/ph18071019

**Published:** 2025-07-09

**Authors:** Ajna Hamidovic, John Davis

**Affiliations:** 1 College of Pharmacy, University of Illinois Chicago, Chicago, IL 60607, USA; 2 School of Medicine, University of Illinois Chicago, Chicago, IL 60607, USA; davisjm@uic.edu

**Keywords:** delirium, olanzapine, atypical antipsychotics, ICU, critical care

## Abstract

**Introduction**: Delirium is a common and debilitating clinical complication among ICU patients. Despite the prevalence of this condition, there are insufficient data to support or refute the routine use of atypical antipsychotics since the existing evidence remains sparse and inconclusive. The objective of the present study was to evaluate whether pre-ICU administration of the atypical antipsychotic olanzapine is associated with a differential time to delirium resolution relative to the control condition. **Methods**: In this emulated clinical trial, we utilized the MIMIC-IV v3.1 database, which contains deidentified health records from approximately 65,000 ICU patients, to derive a cohort of patients with a positive delirium screening within 24 h of ICU admission. We exluded patients who received any antipsychotic other than olanzapine prior to ICU admission. We performed propensity score matching using logistic regression and nearest-neighbor matching (1:1, caliper = 0.2) to balance covariates between the olanzapine and control groups. The primary outcome was time to delirium resolution, defined as the first negative delirium assessment. A Cox proportional hazards model, adjusted for multiple covariates and incorporating age as a time-dependent variable, was used to examine the association between olanzapine use and delirium resolution. Interaction terms were included to evaluate effect modification by age and gender. **Results**: A total of 5070 patients with a positive delirium screening within 24 h and no exposure to other antipsychotics met the eligibility criteria; 421 olanzapine users were matched to 421 controls using propensity score matching. Covariate balance was achieved (all standardized mean differences < 0.1), and no multicollinearity was detected (all VIFs < 2). Pre-ICU olanzapine use was associated with a 27% decrease in the likelihood of delirium resolution (HR = 0.73; 95% CI: 0.63–0.86; *p* < 0.001). A significant interaction with age indicated that the negative impact of olanzapine on delirium resolution increased with advancing age (HR = 1.0024 per unit of age × log(time), *p* = 0.023), translating to a 2.4% increase in the risk of prolonged delirium resolution for every 10-year increase in age per log(time). There was no modification of the association according to gender. **Discussion:** The negative effect of olanzapine on ICU delirium resolution, particularly among the elderly, presented in this study is in line with the results of our earlier study showing a negative effect (i.e., prolonged ICU stay) among patients receiving quetiapine relative to both control and haloperidol conditions. Distinctly strong anticholinergic effects of both olanzapine and quetiapine relative to the other antipsychotic agents may be driving the identified negative outcomes. **Conclusions:** Results of this emulated clinical trial do not support the use of olanzapine for the treatment of ICU delirium because the agent prolongs time to resolution of the condition.

## 1. Introduction

Delirium is a disruption of attention and awareness that develops over a short period of time, typically hours to days. It varies across different settings, but in the intensive care unit (ICU), delirium impacts at least 30% of patients [1]. This debilitating condition is accompanied by at least one additional cognitive disturbance, such as memory deficits, disorientation, language impairment, or perceptual disturbances, and cannot be better explained by a preexisting neurocognitive disorder [2].

Though the median duration of delirium in the ICU is 4 days, the condition can last less than 24 hours or greater than 2 weeks [3,4]. Indeed, time to delirium resolution in the ICU is a robust clinical endpoint as it aligns with enhanced research rigor and patient-centered care. This measure is highly correlated with other outcomes as a longer delirium duration is associated with an increased length of ICU stay, higher mortality, long-term cognitive impairment, and functional decline [5]. Compared with a cross-sectional, binary outcome, such as the presence or absence of delirium at a single time point, the measure of delirium persistence over time is more statistically robust and informative because time-to-event analyses account for both the occurrence and duration of the condition.

Current strategies for treating ICU delirium remain profoundly insufficient. Indeed, the Society of Critical Care Medicine panel was unable to issue a definitive recommendation regarding routine use of antipsychotics for the prevention or treatment of delirium among adult ICU patients in the 2025 focused update to the Pain, Agitation/Sedation, Delirium, Immobility, and Sleep Disruption in Adult Patients in the ICU (PADIS) guideline [6]. Among the eight randomized controlled trials evaluating the efficacy of antipsychotics for the treatment of delirium in the ICU that the panel identified, haloperidol was administered in six, while data on atypical antipsychotics—such as olanzapine, quetiapine, or risperidone—were found to be sparse and heterogeneous, preventing any conclusions to be drawn about their efficacy. Regarding haloperidol, the overall conclusion was that the treatment does not provide any benefit in reducing time to delirium resolution, ICU length of stay, hospital length of stay, or duration of mechanical ventilation, though there may be limited evidence (i.e., low certainty) that it decreases mortality and increases the number of delirium-free days. 

The sparsity of research examining the use of atypical antipsychotics for the treatment of delirium in the ICU underscores the need for more targeted research. Hence, the objective of the present study was to investigate whether pre-ICU administration of olanzapine is associated with a differential time to delirium resolution relative to the control condition (i.e., no olanzapine administration). We utilized an emulated clinical trial framework that approximates the structure and rigor of a randomized controlled trial by carefully defining eligibility criteria, treatment strategies, and analytic approaches.

## 2. Results

### 2.1. Participant Flow

**Figure 1:**
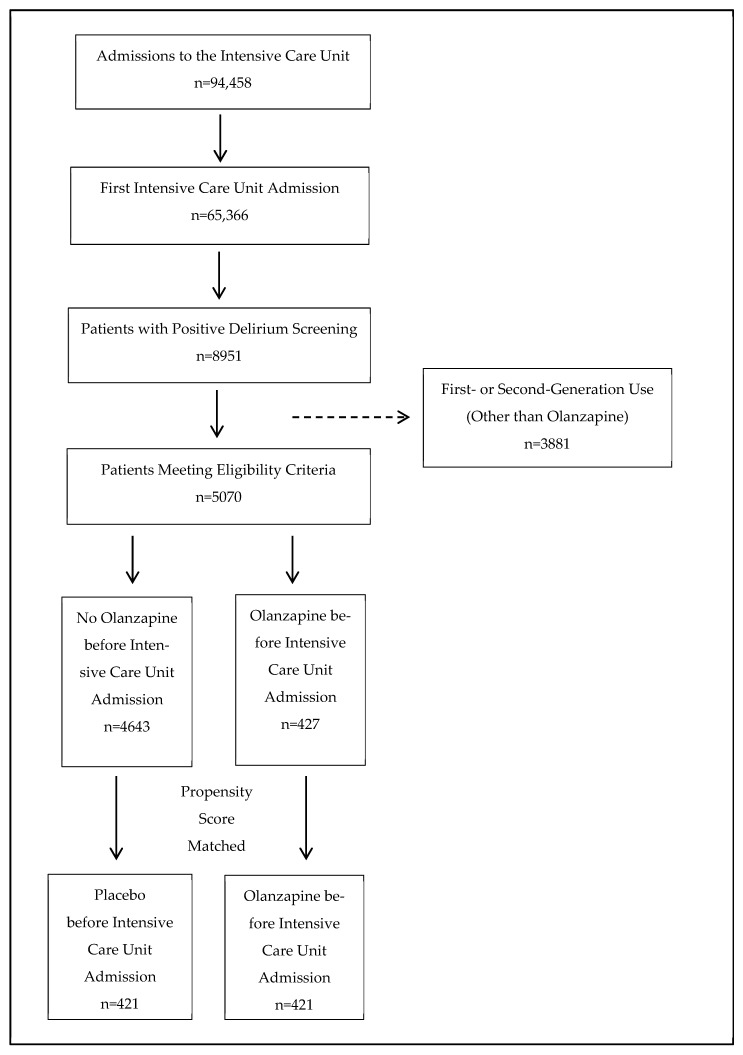
Participant flow diagram.

**Figure 2:**
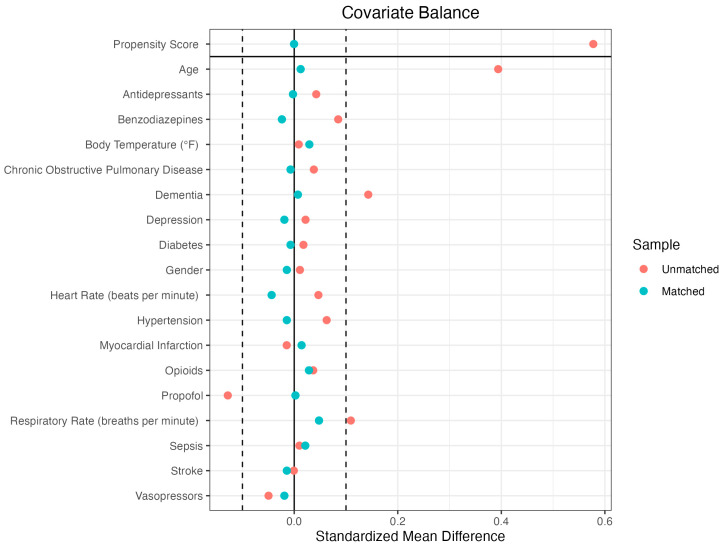
Covariate balance in the unmatched and matched treatment groups.

Figure 1 outlines the cohort selection process for this emulated clinical trial evaluating olanzapine use prior to ICU admission. From an initial 94,458 ICU admissions in the MIMIC database, 65,366 first-time ICU admissions were identified. Among these, 8951 patients had a positive delirium screening within 24 h of ICU admission. After excluding patients who received first- or second-generation antipsychotics other than olanzapine (n = 3881), a total of 5070 patients met the final eligibility criteria. Of these, 427 received olanzapine prior to ICU admission and 4643 did not. For a comparative analysis that includes patients with complete datasets, a propensity score–matched sample was generated, resulting in 421 patients in both the olanzapine and control groups (see Table 1 and Figure 2 for the propensity score matching results and covariate balance).

### 2.2. Propensity Score Matching

The variance inflation factor (VIF) values for all covariates were below 2, indicating no evidence of problematic multicollinearity. Therefore, all variables were retained for propensity score estimation. Following the propensity score matching procedure, 421 cases were matched to 421 controls, resulting in a matched sample of 842 individuals. Prior to matching, the overall distance between the groups was 0.5073. Several covariates displayed substantial imbalances, including age (SMD = 0.43), dementia (SMD = 0.33), and hypertension (SMD = 0.13). Following the matching procedure, the overall distance was substantially reduced to −0.0003, confirming successful alignment of the treated and control cohorts for subsequent analyses. All the covariates achieved an adequate balance of absolute SMDs below the widely acceptable 0.1 value [7]. For results of the propensity matching procedure, please see Figure 2 and Table 1.

### 2.3. Cox Proportional Hazard Analysis

In a time-varying Cox proportional hazards model evaluating predictors of time to delirium resolution among 842 matched ICU patients (600 events), pre-ICU olanzapine use was significantly associated with delayed delirium resolution, with a 27% reduction in the hazard of resolution (HR = 0.73; 95% CI: 0.63 to 0.86; *p* < 0.001) (Table 2). Other factors associated with significantly delayed resolution included propofol use (HR = 0.60; *p* < 0.001)**,** sepsis (HR = 0.71; *p* = 0.0018)**,** stroke (HR = 0.77; *p* = 0.009)**,** and vasopressor use (HR = 0.76; *p* = 0.0039)**.** Female gender was also associated with delayed resolution (HR = 0.81; *p* = 0.016), while dementia approached statistical significance (*p* = 0.056). Other covariates—including diabetes and baseline vital signs—were not significantly associated with time to resolution. The time-varying effect of age (modeled as age × log(time)) was also nonsignificant. Model diagnostics indicated good performance (concordance = 0.625), and global tests confirmed overall model significance (likelihood ratio test *p* < 0.001).

**Figure 3:**
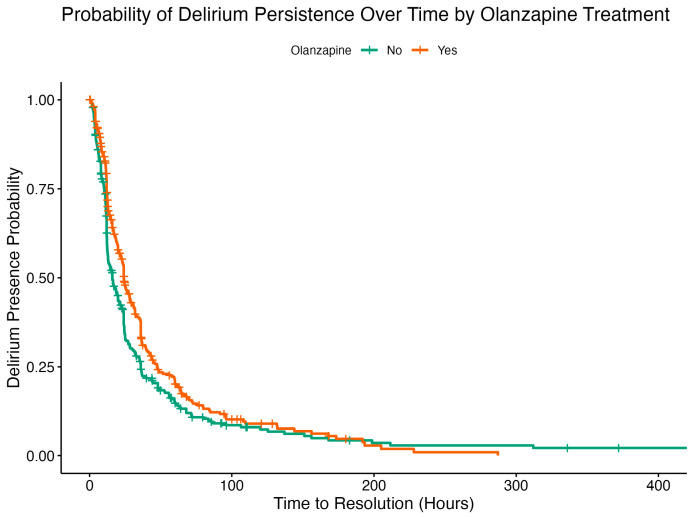
Kaplan–Meier curves for delirium persistence probability according to treatment groups.

The Kaplan–Meier survival curve (Figure 3) depicts the probability of continued delirium (i.e., delirium persistence) over time among ICU patients stratified by pre-ICU olanzapine exposure. Patients who received olanzapine prior to ICU admission (orange line) had a lower probability of delirium resolution at nearly all time points compared to those who did not receive olanzapine (green line). Although the initial drop in both curves suggests that most delirium resolved relatively quickly, the slower decline and prolonged tail of the orange curve indicates that olanzapine exposure was associated with delayed resolution of delirium. The green curve continues further on the *x*-axis, reflecting that more patients in the non-olanzapine group remained under observation at later time points, either due to slower censoring or longer follow-up. This visual finding aligns with the Cox regression results, which showed a significantly reduced delirium resolution in the olanzapine group.

### 2.4. Subgroup Analysis

**Figure 4:**
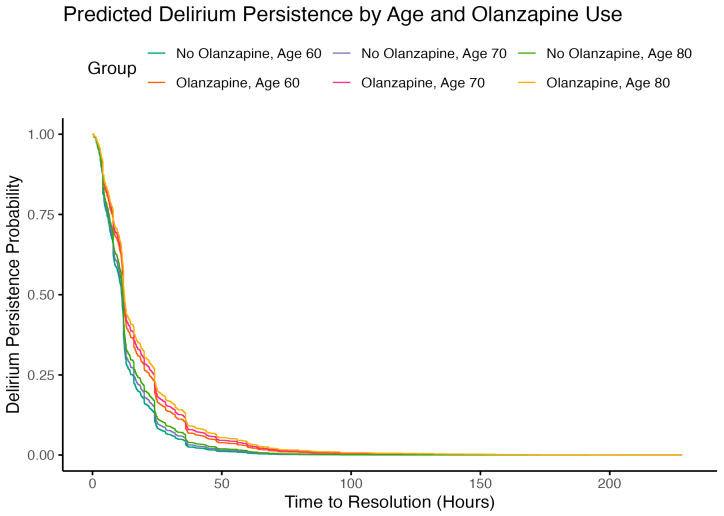
Kaplan–Meier curves for delirium persistence probability according to age and treatment groups.

The effect of olanzapine varied significantly by age over time**,** as indicated by a positive interaction between olanzapine and age (HR = 1.0024 per unit of age × log(time), *p* = 0.023), meaning that the negative impact of olanzapine on delirium resolution becomes more pronounced with increasing age (Figure 4). This translates to a 2.4% increase in the risk of prolonged delirium resolution for every 10-year increase in age per log(time). There was no evidence of effect modification by gender (olanzapine × gender interaction: HR = 1.08, *p* = 0.61).

## 3. Discussion

We report that the pre-ICU administration of olanzapine results in a 27% increase in the risk of prolonged delirium in ICU patients. This finding was more pronounced in the elderly, with a 2.4% increase in the risk of prolonged delirium resolution for every 10-year increase in age per log(time). Based on these results, we emphasize the importance of careful antipsychotic selection—in the elderly in particular—and provide support for deprescribing strategies aimed at minimizing anticholinergic burden prior to ICU admission.

Olanzapine is an atypical antipsychotic with a unique mechanism of action. Its therapeutic effects are linked to the antagonism of D_2_ and 5-HT_2_A receptors. Though this receptor binding profile contributes to its efficacy while in parallel minimizing extrapyramidal side effects, the medication also exhibits a strong binding to the muscarinic acetylcholine M_1_ through M_5_ receptors which contributes to its anticholinergic side effects.

**Figure 5:**
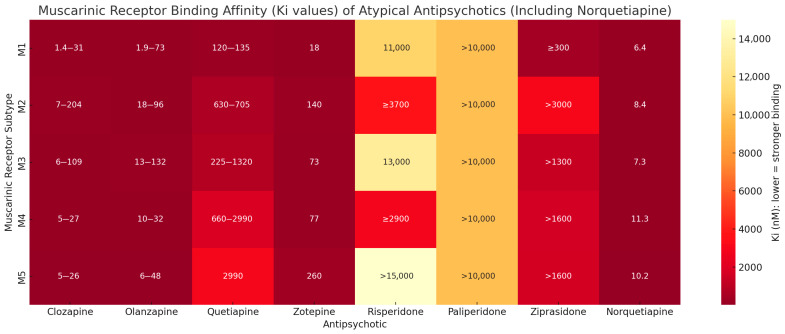
Binding affinity for muscarinic receptor subtypes for seven atypical antipsychotic and norquetiapine, a metabolite of quetiapine.

Relative to the other atypical antipsychotics (Figure 5; generated based on [8,9,10,11]), clozapine exhibits the strongest affinity for muscarinic receptors, with Ki values ranging from 1.4 to 204 nM across all receptor subtypes. Olanzapine also demonstrates substantial binding affinity, with Ki values of 1.9–73 nM at M1, 18–96 nM at M2, 13–132 nM at M3, 10–32 nM at M4, and 6–48 nM at M5. In contrast, quetiapine shows markedly weaker binding, especially for M2 to M5 subtypes, where Ki values exceed 600 nM; however, its active metabolite, norquetiapine, (last section, Figure 5) demonstrates considerably stronger binding across muscarinic receptors, particularly to the M1 (6.4 nM) and M4 (11.3 nM) subtypes. Zotepine presents moderate binding affinity to muscarinic receptors, especially M1 (18 nM), but weaker binding to other subtypes. Risperidone and paliperidone have poor affinity across all muscarinic subtypes, with Ki values generally exceeding 10,000 nM, indicating negligible binding. Ziprasidone shows weak to moderate binding (≥300 to >3000 nM).

Antagonism of distinct muscarinic receptor subtypes results in differential therapeutic and adverse effect profiles. The function of the M1 receptor may be of critical importance in ICU delirium management. Gq/11-coupled, it is densely expressed in the cortex and hippocampus where it modulates cognitive processing, including attention, learning, and memory [10]. On the cellular level, the receptor enhances neuronal excitability via intracellular calcium signaling, thereby facilitating synaptic plasticity and long-term potentiation (LTP) [11,12]. Its blockade or knockout has detrimental effects and results in associative learning task deficits [13]. The remaining M2-M5 subtypes do not seem to contribute to the cognitive deficits—the M2 and M4 receptors regulate tachycardia and possibly extrapyramidal symptoms [14], the M3 receptor mediates peripheral anticholinergic effects such as dry mouth, constipation, urinary retention, and blurred vision [12], while the M5 receptor is implicated in reward processing, cerebral blood flow regulation, and potentially substance use behavior [15].

The antagonism of the M1 receptor is, hence, clinically relevant in the selection of antipsychotics for the treatment of delirium. In fact, we have recently applied Bayesian generalized additive modeling methodology to assess the length of ICU stay among patients with delirium treated with quetiapine, demonstrating that quetiapine administration prolongs ICU stay relative to the control [16]. As both olanzapine and norquetiapine have distinctly strong anticholinergic effects, we hypothesize that their unfavorable effects in the management of delirium are due to this particular mechanism.

In the present study the association between the risk of prolonged delirium and olanzapine administration was particularly pronounced in the elderly. This population is at a higher risk for cholinergic disruption, in large part due to diminished connectivity that affects association systems and cholinergic fibers [17]. Adding to this risk is the finding that patients in the ICU are at a higher risk for delirium, in part due to impaired mobility [18,19]. Hence, elderly patients with delirium who are administered olanzapine (or quetiapine) in the ICU essentially have three sources of increased anticholinergic burden: their age-related pathophysiology, factors related to the ICU stay, and high M1 receptor binding of olanzapine and norquetiapine.

Unfortunately, at this time, treating ICU delirium with antipsychotics does not offer any benefit, and, depending on the specific medication, may, in fact, result in harm. Randomizing 566 critically ill adults with acute delirium in the ICU (as determined by CAM-ICU) to haloperidol, ziprasidone, and placebo, the investigators of the MIND-USA (Modifying the Impact of ICU-Associated Neurological Dysfunction-USA) reported that neither antipsychotic resulted in a reduction in delirium or coma duration compared to placebo. Hence, while studies to date generally showed no benefit of haloperidol in delirium management [6], results presented here caution against the use of olanzapine. We concluded the same for quetiapine based on our earlier analysis [16].

Results of the prior studies on the use of olanzapine for the treatment of ICU delirium have been integrated in a meta-analysis by Liu et al. (2023) [20] who evaluated data from relatively small four randomized controlled trials and six retrospective cohort studies. Their findings showed that olanzapine provided no advantage in alleviating delirium symptoms or shortening delirium duration compared to other pharmacologic interventions as there were no significant differences in ICU or hospital length of stay, in-hospital mortality, or other adverse events. Importantly, the authors noted that the number of available studies was insufficient to support a direct comparison between olanzapine and no intervention, limiting their ability to evaluate the drug’s effectiveness relative to a true control condition. In contrast, the present study utilized an emulated clinical trial design using real-world ICU data to specifically examine the association between pre-ICU olanzapine use and time to delirium resolution relative to a no-antipsychotic control group. Unlike the meta-analysis, which primarily concluded that olanzapine offered no therapeutic advantage, the present study provides direct patient-level evidence that olanzapine, in fact, prolongs delirium. This harmful association was particularly pronounced in older patients, as demonstrated by a significant time-dependent interaction with age—an effect not explored in the prior analysis. Thus, while both studies caution against the use of olanzapine for ICU delirium, the present study advances the evidence by identifying a potential risk of harm rather than merely a lack of benefit.

The present study had limitations and strengths. Though we used only a single center MIMIC database, which limits generalizability, the dataset is validated, robust and well-curated. There are important variables that are not available in the dataset. For example, baseline cognitive status and sleep quality may result in residual confounding despite our robust propensity score matching procedure. Though the patients were identified using the CAM-ICU routine clinical assessment, which enhances the external validity of real-world ICU practice, the measure is binary and does not assess the severity of delirium or provide data on hypoactive and hyperactive subtypes. It is important to note that our findings pertain specifically to pre-ICU olanzapine use and should not be generalized to other atypical antipsychotics. Future research is needed to explore whether similar associations exist for other agents within this chemical class.

We deem our study novel as it addresses a critical gap in the literature by evaluating the impact of pre-ICU olanzapine use on delirium outcomes—a factor that has been largely overlooked in both randomized clinical trials and real-world studies. Importantly, we demonstrate an age-dependent exacerbation of olanzapine’s negative effects, offering the first evidence that elderly patients are especially vulnerable to delayed delirium resolution in this context. By integrating receptor pharmacology into our interpretation, we further highlight that olanzapine’s high muscarinic M1 receptor binding affinity likely contributes to its harmful effects on delirium recovery. Collectively, this study fills a critical gap in the literature, informs clinical practice, and underscores the importance of minimizing anticholinergic burden in ICU patients, especially the elderly.

Future studies should be prospective and conducted in a multicenter fashion, with the stratification of study participants performed according to the delirium subtype. Based on the research findings from our group, we stress the importance of incorporating a measure of anticholinergic burden in their design.

## 4. Materials and Methods

### 4.1. Data Source

The data were sourced from version 3.1 of the Medical Information Mart for Intensive Care (MIMIC)-IV database, which contains health records of approximately 65,000 ICU patients treated at Beth Israel Deaconess Medical Center in Boston, Massachusetts, USA, between the years 2008 and 2022. The database was developed by the Massachusetts Institute of Technology Lab for Computational Physiology in collaboration with the Beth Israel Deaconess Medical Center. Deidentified data are publicly available on PhysioNet upon completion of required training and acceptance of the Data Use Agreement. The release of the deidentified data was approved by the Beth Israel Deaconess Medical Center and the Massachusetts Institute of Technology Institutional Review Boards, with a waiver of individual patient informed consent.

### 4.2. Study Design

Merging data from various modules by integrating patient identifiers and hospital stay information, we formed a cohort of patients based on a positive delirium screening within 24 h of ICU admission. The nursing staff completed the screening at least two times per day using the Confusion Assessment Method for the ICU (CAM-ICU) [21]. The assessment includes four components: (1) an acute change in mental status or fluctuating course, (2) inattention, (3) disorganized thinking, and (4) altered level of consciousness. A positive CAM-ICU assessment is made when a patient exhibits the first and second features, along with either the third or fourth feature. The assessment is recorded as “positive”, “negative”, or “unable to assess”.

Only patients’ first hospital admission data was used in the event a patient was admitted multiple times. Patients receiving an antipsychotic other than olanzapine were excluded. The cohort with a positive CAM-ICU screen within 24 h of admission was then stratified based on the receipt of olanzapine (i.e., olanzapine == 1 and control == 0). The study outcome measure was the time to delirium resolution (i.e., first negative CAM-ICU screening). We collected the following factor (0 == no; 1 == yes) variables: antidepressant administration, benzodiazepine administration, chronic obstructive pulmonary disease, dementia, depression, diabetes, hypertension, myocardial infarction, opioid administration, propofol administration, sepsis, stroke, and vasopressor administration. Additionally, we collected baseline heart rate, respiratory rate, and temperature, as well as age and gender. Given the low level of missingness (<2%), patients with missing data were excluded.

### 4.3. Statistical Analysis

The variance inflation factors (VIFs) were used to examine the presence of multicollinearity among variables. The MatchIt [22] package in R was used to propensity match the strata and achieve covariate balance between the treatment (olanzapine) and control groups. Propensity scores were assigned using a logistic regression model, with the treatment variable as the outcome and the following covariates: age, antidepressant administration, baseline heart rate, baseline respiratory rate, baseline temperature, benzodiazepine administration, chronic obstructive pulmonary disease, dementia, depression, diabetes, gender, hypertension, myocardial infarction, opioid administration, propofol administration, sepsis, stroke, and vasopressor administration. The nearest-neighbor matching without replacement at a 1:1 ratio and a caliper of 0.2 standard deviations of the logit of the propensity score was implemented. Model validity was assessed by calculating standardized mean differences (SMDs), with SMD < 0.1 indicative of a balanced distribution of confounding factors between groups.

A Cox proportional hazards model was used to examine the association between time to delirium resolution and olanzapine. The model included fixed covariates for olanzapine and all the covariates included in propensity matching. Age was incorporated as a time-dependent variable using an interaction with the logarithm of time [i.e., age × log(time)] to capture changing hazard over time. Robust standard errors were estimated by clustering on the matching subclass to account for the matched design. The proportional hazards assumption was evaluated using Schoenfeld residuals and visual inspection of log(-log[survival]) curves [23]. Model fit was further assessed by examining deviance residuals and overall concordance. All analyses were conducted using the coxph function in the R survival package.

To evaluate whether the effect of pre-ICU olanzapine use on delirium resolution varied by patient age and gender, we estimated a Cox proportional hazards model incorporating interaction terms. We included interaction terms for olanzapine × gender and olanzapine × age, with age modeled as a time-varying covariate using a log(time)-dependent transformation: tt(age) = age × log(time). This allowed the age effect, and its interaction with olanzapine, to vary dynamically over time. The model was adjusted for the same set of covariates as in the original Cox model. To account for clustering due to matching, robust standard errors were estimated using the cluster() option by subclass. The *coxph()* function from the survival package in R was used to fit the model.

## 5. Conclusions

In conclusion, the results of this study show a significantly increased risk of delirium persistence in ICU patients receiving olanzapine. The present study not only advances clinical practice but also prioritizes the importance of cholinergic function preservation in ICU delirium patients.

## Figures and Tables

**Table 1:** Standardized mean difference (SMD) according to treatment group before and after propensity score matching.

	Before Matching			After Matching		
	Means Treated	Means Control	Standardized Mean Difference	Means Treated	Means Control	Standardized Mean Difference
Propensity Score	0.1145	0.0827	0.5036	0.1145	0.1144	0.0008
* Demographics*						
Age	72.9739	66.5803	0.4264	72.9739	72.8195	0.0103
Gender	0.2138	0.1713	0.1037	0.2138	0.2043	0.0232
* Medical Conditions*						
Chronic Obstructive Pulmonary Disease	0.1306	0.0927	0.1125	0.1306	0.152	−0.0634
Dementia	0.247	0.104	0.3316	0.247	0.247	0
Depression	0.2067	0.1848	0.054	0.2067	0.1876	0.0469
Diabetes	0.3302	0.3123	0.0379	0.3302	0.3325	−0.0051
Hypertension	0.5463	0.4836	0.126	0.5463	0.5487	−0.0048
Myocardial Infarction	0.133	0.1475	−0.0427	0.133	0.1686	−0.1049
Sepsis	0.2518	0.2418	0.023	0.2518	0.2755	−0.0547
Stroke	0.2043	0.205	−0.0017	0.2043	0.1781	0.0648
* Medications*						
Antidepressants	0.2138	0.1713	0.1037	0.2138	0.2043	0.0232
Benzodiazepines	90.304	89.2533	0.0499	90.304	92.3539	−0.0973
Propofol	20.3183	19.6346	0.1105	20.3183	20.5701	−0.0407
Vasopressors	98.2389	98.2224	0.011	98.2389	98.1964	0.0284
* Vitals*						
Heart Rate	90.304	89.2533	0.0499	90.304	92.3539	−0.0973
Respiratory Rate	20.3183	19.6346	0.1105	20.3183	20.5701	−0.0407
Body Temperature	98.2389	98.2224	0.011	98.2389	98.1964	0.0284

**Table 2:** Summary of the Cox proportional hazards analysis.

Term	Estimate	SE	Robust SE	Statistic	*p* Value	Confidence-Low	Confidence-High
* Demographics*							
Age	0.9985	0.0011	0.0011	−1.4209	0.1553	0.9963	1.0006
Gender (F)	0.8086	0.0865	0.0881	−2.4111	0.0159	0.6803	0.9610
* Medications*							
Olanzapine	0.7342	0.0832	0.0792	−3.9005	0.0001	0.6287	0.8575
Antidepressants	0.8813	0.1086	0.0934	−1.3525	0.1762	0.7338	1.0584
Benzodiazepines	0.9023	0.0888	0.0871	−1.1802	0.2379	0.7606	1.0703
Propofol	0.6030	0.0962	0.0909	−5.5614	0.0000	0.5046	0.7207
Vasopressors	0.7571	0.1020	0.0964	−2.8872	0.0039	0.6268	0.9145
* Vitals*							
Heart Rate	0.9971	0.0021	0.0020	−1.4751	0.1402	0.9933	1.0010
Respiratory Rate	0.9882	0.0075	0.0078	−1.5135	0.1301	0.9732	1.0035
Body Temperature	0.9978	0.0317	0.0312	−0.0693	0.9447	0.9387	1.0607
* Medical Conditions*							
Chronic Obstructive Pulmonary Disease	0.9984	0.1230	0.1278	−0.0122	0.9903	0.7772	1.2827
Dementia	0.8108	0.1122	0.1096	−1.9137	0.0557	0.6541	1.0051
Depression	1.0512	0.1085	0.0949	0.5257	0.5991	0.8727	1.2662
Diabetes	0.8728	0.0903	0.0837	−1.6259	0.1040	0.7407	1.0284
Hypertension	0.9752	0.0846	0.0812	−0.3089	0.7574	0.8317	1.1435
Myocardial Infarction	1.0104	0.1379	0.1351	0.0765	0.9390	0.7754	1.3166
Sepsis	0.7070	0.1115	0.1112	−3.1188	0.0018	0.5686	0.8791
Stroke	0.7668	0.1045	0.1015	−2.6148	0.0089	0.6284	0.9357

## Data Availability

Restrictions apply to the availability of these data. Data were obtained from physionet.org and are available from physionet.org with the permission of physionet.org.
